# Risk Factors for Early Cholangitis Following Biliary Plastic Stent Placement

**DOI:** 10.1055/a-2904-9716

**Published:** 2026-07-23

**Authors:** Bianca Doamna, Anca Sugeac, Cristiana Popp, Andreea Benguş, Georgiana Stoian, Theodor Alexandru Voiosu, Bogdan Mateescu, Radu Voiosu, Andrei Mihai Voiosu

**Affiliations:** 1 87267 Carol Davila University of Medicine and PharmacyBucharestRomania; 2 Gastroenterology 433892 Colentina HospitalBucharestBucharestRomania; 3 Pathology 433892 Colentina HospitalBucharestBucharestRomania

**Keywords:** endoscopic retrograde cholangiopancreatography, biliary plastic stent, cholangitis, bile culture, stent occlusion, biliary drainage

## Abstract

**Background and study aim** Biliary plastic stents are widely used for biliary drainage but are prone to dysfunction and causing cholangitis. The aim of this study was to identify the risk factors for early post-ERCP cholangitis in patients with biliary plastic stents, assess clinical impact, and evaluate changes in bile composition associated with indwelling stents.

**Patients and methods** We conducted a prospective, longitudinal observational study including consecutive patients with native papilla who underwent endoscopic retrograde cholangiopancreatography (ERCP) with biliary plastic stent placement at a tertiary referral center. Bile was aspirated at index ERCP prior to contrast injection and processed for microbiologic and chemical analysis. A follow-up procedure was scheduled at 3 months when we retrieved the stent for patency analysis and obtained a new bile sample. The primary outcome was early cholangitis after the index procedure (<3 months). Secondary outcomes included bile culture positivity, bile composition changes, stent occlusion degree, and cholangitis-related mortality. Multivariable binary logistic regression was used to identify independent predictors of early cholangitis, with results expressed as odds ratios (ORs) with 95% confidence intervals.

**Results** A total of 159 patients were included, with malignant strictures accounting for 84.4% of indications. Complete follow-up was available for 136 patients, of whom 47 (34.6%) developed early cholangitis after a median of 32.5 days. On multivariable analysis, a positive bile culture at index ERCP was the only independent predictor of early cholangitis (OR 4.71, 95% CI 1.69–13.17). Degree of stent occlusion, stent characteristics, and bile duct obstruction severity were not associated with cholangitis. Seven deaths were directly attributable to cholangitis.

**Conclusions** Early cholangitis after biliary plastic stenting is frequent and associated with positive bile cultures at index ERCP. Bile culture obtained at stent placement may help identify patients at increased risk of early cholangitis.

## Introduction

Endoscopic placement of plastic stents (PSs) in the biliary ducts was introduced more than 40 years ago and is still being routinely used to ensure bile drainage in benign and malignant strictures, postoperative fistulas, and in some cases of complex choledocholithiasis. 1 While PSs are effective for initial biliary decompression, stent dysfunction mainly caused by occlusion is common 2 3 4 and can lead to life-threatening cholangitis. As self-expandable metal stents have longer patency, a lower rate of early dysfunction, and the need for reintervention, current guidelines strongly recommend metal stenting over plastic stenting in most instances of confirmed malignancy, either as definitive palliation or preoperatively for pancreatic tumors. 5

However, biliary PSs are still frequently employed across a wide variety of indications, both benign and malignant, so understanding the mechanisms and risk factors for early occlusion is a clinically relevant research area. PS extraction or exchange is suggested at 3 months in order to prevent cholangitis, but there is a lack of high-quality evidence to sustain this recommendation. 6 7 Furthermore, early cholangitis occurring before scheduled revision is frequent in clinical practice and constitutes a serious and life-threatening event while also considerably burdening emergency endoscopy services.

The aim of this prospective study was to identify the risk factors for and outcomes of early post-ERCP cholangitis in patients with biliary plastic stents.

## Patients and Methods

### Study Design

The TEMPEST study was a prospective, longitudinal, observational study enrolling consecutive patients with native papilla who underwent biliary plastic stent placement during ERCP between September 2020 and August 2022 at our tertiary referral unit. The study presupposed data collection at two points: the index and the follow-up ERCP and telephonic contact during the follow-up. Interim results were presented at the annual meeting of the European Society for Gastrointestinal Endoscopy. 8

### Patient Population

All patients aged 18–90 years with native papilla who were admitted for ERCP were screened for inclusion, and informed consent was obtained prior to the procedure. Patients were included only if placement of biliary PS for any indication was performed. We excluded patients with prior surgical or endoscopic hepatobiliopancreatic interventions such as biliary derivations, previous sphincterotomy, or biliary stenting.

As a referral center for biliopancreatic diseases, our unit receives mostly patients with complex and advanced disease, which accounts for the high percentage of malignant indications and rates of cholangitis at presentation from our cohort.

Initial assessment included standard clinical examination, blood work, and imaging studies as clinically required. Patients were classified according to the Tokyo Guidelines for Acute Cholangitis (TG18) 9 and American Society of Anesthesiologists Physical Status (ASA PS) classification system. 10 Data was collected in a standardized study form and introduced in an anonymized electronic database. Recent use of medication potentially relevant to the study (i.e., antibiotics, ursodeoxycholic acid, proton pump inhibitors, chemotherapy) was noted. All patients received standard of care treatment according to institutional and national guidelines.

### Visit #1: Index ERCP Procedure

ERCP was performed according to our institutional protocol with standard ERCP prophylactic measures, 11 under propofol sedation or intubation where needed. For biliary cannulation, a triple-lumen sphincterotome (Ultratome, Boston Scientific) with a preloaded 0.035″ guidewire was used for initial attempts at cannulation, with adjunctive techniques such as double guidewire or precut techniques employed at the discretion of the attending endoscopist. We recorded essential procedural steps such as technique for cannulation, instances of difficult biliary cannulation, and need for different techniques (especially injection prior to cannulation).

#### Biliary Sampling

We routinely flushed the sphincterotome lumen with 5 mL saline prior to insertion in the biliary ducts to avoid bacterial contamination as much as possible. After deep biliary cannulation, we first aspirated bile for analysis and culture using a sterile 10 mL syringe, prior to any contrast injection, to minimize contamination or dilution. We aimed to collect approximately 3–5 mL of bile, which was immediately transferred to sterile containers. Samples were transported to the microbiology laboratory within 30 min of collection. Bile culture was performed in all patients from whom bile could be aspirated ( *n* = 152 at index ERCP; *n* = 56 at follow-up ERCP). Sufficient bile volume for biochemical composition analysis (IgG, IgA, total protein, cholesterol; minimum ~3 mL) was obtained in a subset of these patients ( *n* = 140 at index ERCP; *n* = 41 at follow-up ERCP).

Bile cultures were processed on both solid aerobic media (blood agar and MacConkey agar) and in anaerobic enrichment broths to support the growth of both aerobic and anaerobic organisms. Bacterial identification was performed using an automated VITEK MS or VITEK 2 COMPACT system (bioMérieux), based on the morphological appearance and Gram staining of isolated colonies. Antimicrobial susceptibility testing was carried out using automated microdilution panels specific to the isolated strain. Interpretation of susceptibility results was performed according to CLSI 2024 guidelines. 12 In selected cases involving suspected or confirmed multidrug-resistant organisms (e.g., Enterobacter spp., Acinetobacter spp., Pseudomonas spp., Stenotrophomonas maltophilia), extended panels including reserve antibiotics—such as Cefiderocol—were also applied.

Culture and susceptibility results were made available to the treating clinician and no blinding was applied. Antibiotic therapy was adjusted by the physician, based on the antibiogram, typically within 72 h from the procedure. Postprocedural antibiotic use was systematically recorded.

#### Stenting

The decision to place PSs and their characteristics (length, shape, and diameter) were at the discretion of the attending endoscopist. We used biliary plastic stents with sizes of 7–8.5Fr–10Fr, lengths ranging from 5 to 15 cm, and either Amsterdam/straight duodenal bend or double-pigtail in shape.

Post-procedurally, we performed blood work (complete blood count, lipase, total bilirubin, CRP level) about 6 h after the procedure. Patients were discharged after 24 h if no adverse events occurred. We noted use of medication of interest during admission and at discharge. Adverse events were defined and graded according to the ASGE/AGREE classification system for endoscopic adverse events. 13

In order to minimize the risk of duodenoscope-associated infections, our department adheres to the ESGE guidelines for endoscope reprocessing. 14 The protocol includes pre-cleaning, manual decontamination using a 1% multi-enzymatic detergent (Sekusept), followed by high-level disinfection with 2% Sekusept active for 30 min, with recirculation through all channels. Alternatively, fully automated reprocessing is used (50-minute cycle including decontamination, disinfection, rinsing, and drying). Endoscopes are stored under sterile conditions. Surveillance cultures are performed twice weekly on all duodenoscopes.

### Visit #2

#### Follow-up

We followed up patients through telephone interviews with them or their caregivers at 1 and 2 months to detect events of interest (i.e., fever, chills, pruritus, jaundice, hospital admissions, death). All patients were scheduled to return for stent revision/extraction at 3 months or earlier in case of symptoms suggesting stent dysfunction.

#### Second ERCP and Stent Retrieval

**Fig. 1:**
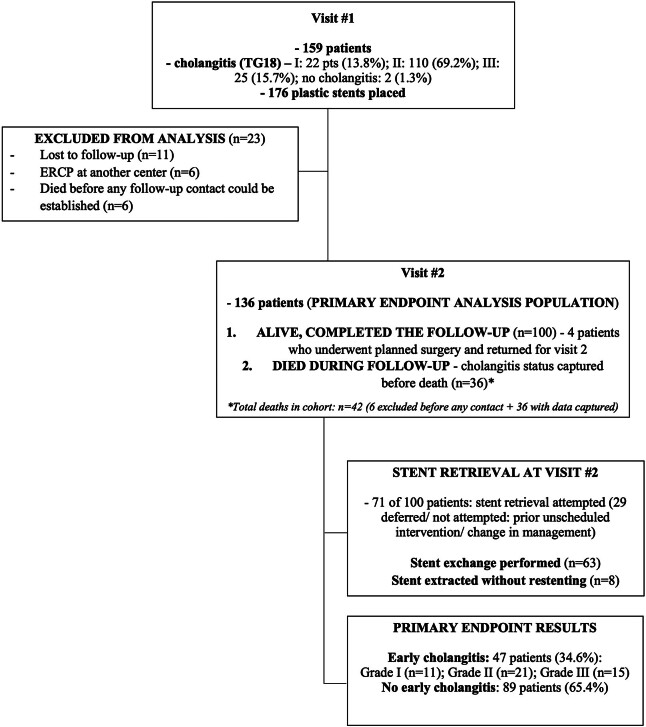
Flowchart of the TEMPEST study. Of the 159 consecutive patients enrolled at index ERCP, 23 were excluded from the primary endpoint analysis: 11 were lost to follow-up, 6 underwent their second ERCP at another center, and 6 died before any follow-up contact could be established. The remaining 136 patients constitute the primary endpoint analysis population. Of these, 100 were alive and attended Visit 2 in person (including 4 patients who had undergone planned surgical intervention between visits but returned per protocol); 36 patients died during the follow-up period but contributed outcome data, as their cholangitis status had been documented prior to death through scheduled telephone contacts or hospital records. Total deaths in the cohort were 42 (26.4% of 159 enrolled): 6 excluded before any contact and 36 with documented outcome data. Of the 100 patients attending Visit 2 in person, stent retrieval was attempted in 71; in 63 a stent exchange was performed and in 8 the stent was extracted without restenting. Retrieval was not attempted or was deferred in the remaining 29 patients. Reasons for failed retrieval in attempted cases included proximal stent migration, distal migration, and impaction. Of the 136 patients in the analysis population, 47 (34.6%) developed early cholangitis (Grade I: *n* = 11; Grade II: *n* = 21; Grade III: *n* = 15) and 89 (65.4%) did not.

At the second visit (either scheduled or emergent), we performed the same work-up as at the index admission. During the second ERCP, we attempted to remove all stents using snares or foreign-body forceps, either through the duodenoscope channel or by removing the endoscope, according to the size and number of the stents. Stent retrieval was not always possible at the second ERCP. Reasons for failed retrieval included proximal stent migration, distal migration, and inability to grasp the stent due to impaction. Cases of failed retrieval are shown in Fig. 1 . After deep biliary cannulation was achieved, a bile sample was obtained and processed in the same fashion as at the initial visit.

Immediately after retrieval, the retrieved stents were first placed in 10% buffered formalin for a duration ranging from 4 to 48 h to ensure proper fixation. Following fixation, the stents were sectioned into 1 mm thick segments using a scalpel. These segments were then mounted on slides using Pertex mounting medium, and coverslips were applied to prepare them for microscopic examination.

The prepared slides were examined under an optical microscope to assess the degree of obstruction at both the intestinal and biliary ends of the stents. This analysis aimed to provide detailed insights into the extent and nature of any blockages present. Upon microscopic examination, the degree of obstruction at both the intestinal and biliary ends of the stents was quantified using image analysis software with a public domain Java image processing program (ImageJ, https://imagej.net/ij/). Cross-sectional microscopic images of the stent lumen were obtained, and the obstructive intraluminal content was manually delineated. The area occupied by the obstruction was then calculated and expressed as a percentage of the total luminal cross-sectional surface, allowing for a semiquantitative assessment of stent occlusion.

Due to the predominance of malignant pathology in patients referred to our service and the lack of integrated electronic reporting, we used telephonic interviews and follow-up visits to try and capture all adverse events of interest (such as emergent hospitalization, documented cholangitis, or death). Planned surgical interventions taking place between the first and second visits (e.g., duodenopancreatectomy for pancreatic ductal adenocarcinoma) were recorded and, whenever possible, the retrieval of the stent was attempted.

### Definitions and Outcomes

Our main outcome of interest was the rate of early cholangitis, defined as a new episode of cholangitis fulfilling TG18 criteria 15 occurring after the resolution of the presenting episode and before the scheduled 3-month stent-exchange visit. Early cholangitis in our study refers to recurrent or new episodes arising following initial biliary drainage and antibiotic treatment, rather than persistence of the index episode. Severity grading (TG18 grade I ( *n* = 11)/ II ( *n* = 21)/ III ( *n* = 15)) was recorded for all follow-up cholangitis episodes. Secondary outcomes included: death attributable to cholangitis, rate of positive bacterial cultures at index and follow-up procedures, changes in bile composition after stenting (IgG, IgA, protein, cholesterol, calcium), and degree of stent lumen occlusion. Cases of cholangitis occurring within 24 h of ERCP were excluded to avoid confounding related to technical errors in stent placement.

We explored various risk factors for early cholangitis in our cohort: benign/malignant indication for stenting; positive bile culture at index and follow-up procedures; degree of occlusion in cases of retrieved stents; changes in bile composition, stent diameter and shape; and use of antibiotics.

### Ethics

This study was approved by the Ethics Committee of Colentina Clinical Hospital and respected the updated Helsinki Declaration of human rights. As the decision to place a biliary PS is sometimes taken intraprocedural (e.g. complex choledocholithiasis, indeterminate stricture), all patients undergoing ERCP were considered for inclusion. In cases where the decision to place a stent had not been anticipated, bile was sampled but only sent for analysis if the patient gave informed consent for inclusion.

### Statistical Analysis

Binary logistic regression was chosen as the primary analytical approach because early cholangitis was defined as a binary outcome (occurrence before the scheduled 3-month visit). Patients who died or were lost to follow-up before the 3-month visit without a documented cholangitis episode were excluded from the primary logistic regression analysis. Time-to-event analyses (Kaplan–Meier, Log-Rank Test) were performed as secondary analyses to describe the temporal distribution of cholangitis events.

Variables included in the multivariable logistic regression model were selected a priori based on clinical relevance and existing literature, regardless of their univariable significance: age, sex, indication for ERCP (benign vs. malignant), total bilirubin at index visit, antibiotic use at discharge, and stent diameter. These variables were considered potential confounders of the association between bile culture positivity and early cholangitis based on prior published data and clinical reasoning. We used Shapiro–Wilk to test normality and report continuous variables as mean and standard deviation (SD) for normal distribution, and median and range if the data were not normally distributed. For categorical variables, we used the chi-square test or Fisher’s exact test as appropriate. Quantitative variables with nonparametric distribution were tested between groups using the Mann–Whitney *U* test. Univariable logistic regression models were used to predict the odds of having antibiotics in the prior week or early cholangitis; performance of the predictions was calculated using odds ratios with 95% confidence intervals along with the significance values. A multivariable logistic regression model was used to assess the effect of having antibiotics in the prior week while adjusting for age, gender, diagnostic, bilirubin value at first visit, usage of antibiotic after the first visit, and stent diameter. The time until development of cholangitis was compared between groups using the Log-Rank Test; description of time was made using medians with interquartile range and Kaplan–Meier curves.

We considered a two-sided *p* -value < 0.05 statistically significant in this analysis. All analyses were performed using IBM SPSS Statistics (v.25).

## Results

### Demographics

In this study, we included 159 patients who underwent their index ERCP procedure and received one or more biliary plastic stents. Ninety-nine (62%) were male, and the mean age for the cohort was 65 ± 11 years. The indications for stenting were: malignant strictures (84.4%), choledocholithiasis (7.5%), benign strictures (5%), and bile duct leaks or other indications (3.1%). According to the TG18 criteria, 135 patients (84.9%) had at least moderate cholangitis at their index visit, and 75.4% had an ASA grade of ≥3. Table 1 shows the main characteristics of the study cohort and Fig. 1 gives the study flow-chart.

**Table 1:** Population characteristics.

Population characteristics	Visit 1 – index ERCP ( *n* = 159)
Age, years, mean Men, *n* (%)	65 99 (62)
Women, *n* (%)	60 (38)
ASA score, *n* (%)	
ASA I	4 (2.5)
ASA II	35 (22)
ASA III	98 (61.6)
ASA IV	22 (13.8)
TG18 criteria for cholangitis, *n* (%)	
No cholangitis	2 (1.3)
Grade I	22 (13.8)
Grade II	110 (69.2)
Grade III	25 (15.7)
Indication for ERCP, *n* (%)	
Malignant stricture	134 (84.3)
Benign stricture	8 (5)
Choledocholithiasis	12 (7.5)
Fistula	4 (2.5)
Other	1 (0.6)
Antibiotic use before index ERCP, *n* (%)	39 (24.5)
BMI (kg/m ^2^ )	25
Total bilirubin (mg/dL) median, IQR (min/max)	14, 8.1–22 (0.17/49)
WBC (×1000/mm ^3^ ), mean (min/max)	9413 (3760/24460)
CRP (mg/L) mean (min/max)	39 (0.40/317)
MDRD, mean (min/max)	78 (3/191)

Abbreviations: ASA, American Society of Anesthesiologists; CRP, C-reactive protein; ERCP, endoscopic retrograde cholangiopancreatography; IQR, interquartile range; TG18, Tokyo Guidelines 2018; MDRD, Modification of Diet in Renal Disease (four-variable equation for estimated glomerular filtration rate); eGFR, estimated glomerular filtration rate.

### ERCP and Stenting

Of the 159 ERCP procedures, biliary access was gained through the guidewire technique in 124 (78%), precut in 22 (13.8%), contrast injection in 10 (6.3%), and double guidewire in 2 (1.3%) cases. The cannulation was judged as difficult in 82 (51.6%) cases, and the pancreatic duct was accessed at least once in 62 (39 %) patients and stented prophylactically in 18 (11.3%). Sphincterotomy was performed in most patients (151, 95%). In total, 176 biliary PSs were placed and 17 patients received two or more biliary stents, mostly for malignant strictures. Bile culture was performed in 152 patients at the index ERCP and 56 at the follow-up procedure. Of these, sufficient bile volume for biochemical composition analysis was available in 140 patients at the index ERCP and 41 at follow-up.

Post-ERCP pancreatitis was registered in 26 patients (16.4%) but was mild (grade I) in most cases ( *n* = 24), as classified by the ASGE/AGREE adverse events severity grading system. 13 The post-ERCP pancreatitis rate of 16.4% observed in our cohort is higher than rates reported in ERCP populations. Difficult biliary cannulation, recorded in 51.6% of our procedures, and inadvertent pancreatic duct access in 39% of patients are well-recognized predictors of PEP and likely account for this elevated rate. Standard prophylactic measures were applied throughout, including rectal NSAIDs per institutional protocol and prophylactic pancreatic stenting in cases judged at high risk (11.3%).

### Follow-up and Early Cholangitis

During the study period, 42 (26.4%) patients died, and 17 (10.6%) were lost to follow-up or underwent ERCP in another unit. The 26% mortality within the follow-up period reflects the advanced disease stage and high morbidity of our cohort (84.4% malignant indications; 75.4% ASA ≥ 3). The causes of death were: progression of underlying malignancy ( *n* = 32), cholangitis-attributable sepsis ( *n* = 7), and postsurgical complications ( *n* = 3). Patients who died were not excluded from the primary endpoint analysis; those who developed cholangitis prior to death were counted, while those who died without a documented prior cholangitis episode were censored at the time of death in the analysis. Complete follow-up data according to our protocol were available for 136 patients of whom 47 (34.6%) developed early cholangitis after a median of 32.5 days (IQR 11.5–69.5).

A total of 26 patients underwent scheduled surgical intervention between visit 1 and visit 2, as part of their planned therapeutic management, most commonly for malignant biliary strictures. These procedures were not performed in response to complications, but rather as anticipated steps in the overall treatment course. Of the 26 patients, only 4 returned for visit 2 and completed full follow-up as per protocol. The remaining 22 patients did not complete visit 2 follow-up, either due to death, transfer to another center, or loss to follow-up.

We identified 7 deaths directly attributable to cholangitis, but overall early cholangitis did not associate with increased risk of death during follow-up ( *p* = 0.903, chi-square).

A positive biliary culture at the index ERCP was the only factor significantly associated with cholangitis during follow-up (OR 3.312, 95% CI 1.406–7.801, *p* = 0.006) ( Table 2 ). On multivariable analysis after adjusting for age, gender, indication for ERCP, initial bilirubin level, and use of antibiotics after the procedure, a positive bile culture at the index ERCP remained the only risk factor for early cholangitis (OR 4.71, 95% CI 1.69–13.17) ( Table 3 ).

**Table 2:** Comparison between patients who developed early post-ERCP cholangitis and those without cholangitis at the follow-up ERCP.

Variable	Cholangitis ( *n* = 47)	No cholangitis ( *n* = 89)	*p* -Value
Age (median, IQR)	67 (63-73)	66 (58.5-72.5)	0.330
Male sex, *n* (%)	34 (72.3)	55 (61.8)	0.219
Malignant indication, *n* (%)	42 (89.4)	72 (80.9)	0.202
Initial bilirubin (μmol/L) (median, IQR)	15 (7.94–21.62)	13.5 (9.05–22)	0.770
Difficult canulation, *n* (%)	22 (46.8)	47 (52.8)	0.506
Post-ERCP pancreatitis, *n* (%)	7 (14.9)	10 (11.2)	0.525
Number of stents placed (median, IQR)	1 (1–1)	1 (1–1)	0.933
Positive bile culture at index ERCP, *n* (%)	16 (34.0)	12 (13.5)	**0.006**

Abbreviations: ERCP, endoscopic retrograde cholangiopancreatography; IQR, interquartile range.

**Table 3:** Univariable and multivariable logistic regression analysis of risk factors for early cholangitis.

Variable	Univariable OR (95% CI)	*p*	Multivariable OR (95% CI)	*p*
Age	1.016 (0.985–1.049)	0.319	1.02 (0.98–1.06)	0.39
Gender	0.619 (0.287–1.334)	0.221	0.49 (0.20–1.19)	0.11
Benign/malignant	1.983 (0.682–5.766)	0.209	2.57 (0.68–9.66)	0.16
Bilirubin at visit #1	1.000 (0.963–1.038)	0.990	1.00 (0.96–1.05)	0.85
**Positive bile culture at visit #1**	**3.312 (1.406–7.801)**	**0.006**	**4.71 (1.69–13.17)**	**0.003**
Antibiotic at discharge	1.975 (0.938–4.159)	0.073	1.90 (0.84–4.72)	0.12
Stent diameter	0.469 (0.164–1.345)	0.159	0.57 (0.18–1.78)	0.33

Importantly, no duodenoscope-related infections were documented during the study.

### Bile Composition Analysis

We were able to sample sufficient bile for analysis in 140 patients at the index ERCP and 41 at the follow-up procedure. We noticed that biliary concentrations of IgG and IgA significantly increased after stenting (median IgA 10.3 (6.7–14.85) vs. 3.5 (1.7–8.45), *p* < 0.001, median IgG 8(3–13.22) vs. 1 (0–2.75), *p* < 0.001, Wilcoxon signed-ranks tests), while biliary cholesterol levels at follow-up were significantly lower in patients with cholangitis (median 20.2 (11.4–59.77) vs. 66.65 (33.2–76.57), *p* = 0.005, Mann–Whitney *U* test).The most frequently isolated bacteria were *Escherichia coli* , *Klebsiella pneumoniae* , and *Enterococcus* spp., with *E. coli* being the most prevalent at both ERCP procedures ( Table 4 ). At index ERCP, *E. coli* showed the highest susceptibility to Piperacillin/Tazobactam (62.8%) and Trimethoprim/Sulfamethoxazole (50%), but both declined substantially at follow-up (32.25% and 16.12%, respectively). *K. pneumoniae* demonstrated persistently low and decreasing susceptibility to Ciprofloxacin and Gentamicin. *Enterococcus* spp. maintained low susceptibility overall, with a notable drop in response to Gentamicin between visits.

**Table 4:** Susceptibility to antibiotics of bacteria cultured from bile samples.

	Positive bile cultures at index ERCP ( *n* = 35/152)						Positive bile cultures at follow-up ERCP ( *n* = 46/56)					
	AMC	CTR	CIP	GEN	TMP/SMX	TZP	AMC	CTR	CIP	GEN	TMP/SMX	TZP
*Escherichia Coli*	5/14 (35.71%)	3/14 (21.42%)	6/14 (42.85%)	4/14 (28.57%)	7/14 (50.00%)	9/14 (62.28%)	4/31 (12.90)	4/31 (12.90)	5/31 (16.12%)	7/31 (22.58%)	5/31 (16.12%)	10/31 (32.25%)
*Klebsiella pneumonia*	3/9 (33.33%)	3/9 (33.33%)	2/9 (22.22%)	3/9 (33.33%)	2/9 (22.22%)	3/9 (33.33%)	1/7 (14.28%)	1/7 (14.28%)	2/7 (28.57%)	3/7 (42.85%)	2/7 (28.57%)	2/7 (28.57%)
*Enterococcus*	0/7 (0%)	0/7 (0%)	1/7 (14.28%)	5/7 (71.42%)	0/7 (0%)	0/7 (0%)	0/2 (0%)	1/2 (50%)	0/2 (0%)	0/2 (0%)	0/2 (0%)	0/2 (0%)
*Candida spp.*	0/1 (0%)	0/1 (0%)	0/1 (0%)	0/1 (0%)	0/1 (0%)	0/1 (0%)	0/0 (0%)	0/0 (0%)	0/0 (0%)	0/0 (0%)	0/0 (0%)	0/0 (0%)
*Stenotrophomonas* *Maltophila*	0/2 (0%)	0/2 (0%)	1/2 (50%)	0/2 (0%)	2/2 (100%)	0/2 (0%)	0/1 (0%)	0/1 (0%)	0/1 (0%)	0/1 (0%)	1/1 (100%)	0/1 (0%)
*Proteus*	1/2 (50%)	2/2 (100%)	0/2 (0%)	0/2 (0%)	0/2 (0%)	1/2 (50%)	1/2 (100%)	0/1 (0%)	0/1 (0%)	0/1 (0%)	0/1 (0%)	0/1 (0%)
*Morganella Morganii*	0/1 (0%)	0/1 (0%)	1/1 (100%)	1/1 (100%)	1/1 (100%)	1/1 (100%)	0/0 (0%)	0/0 (0%)	0/0 (0%)	0/0 (0%)	0/0 (0%)	0/0 (0%)
*Pseudomonas aeruginosa*	0/1 (0%)	0/1 (0%)	1/1 (100%)	1/1 (100%)	0/1 (0%)	1/1 (100%)	0/2 (0%)	0/2 (0%)	1/2 (50%)	1/2 (50%)	1/2 (50%)	1/2 (50%)
*Streptococcus Salivarium*	0/1 (0%)	0/1 (0%)	0/1 (0%)	0/1 (0%)	0/1 (0%)	0/1 (0%)	0/0 (0%)	0/0 (0%)	0/0 (0%)	0/0 (0%)	0/0 (0%)	0/0 (0%)
*Citrobacter freundii*	0/0 (0%)	0/0 (0%)	0/0 (0%)	0/0 (0%)	0/0 (0%)	0/0 (0%)	0/1 (0%)	1/1 (100%)	1/1 (100%)	0/1 (0%)	0/1 (0%)	0/1 (0%)
*Enterobacter cloacae*	0/0 (0%)	0/0 (0%)	0/0 (0%)	0/0 (0%)	0/0 (0%)	0/0 (0%)	0/1 (0%)	0/1 (0%)	1/1 (100%)	0/1 (0%)	0/1 (0%)	1/1 (100%)

Abbreviations: AMC, amoxicillin and clavulanic acid; CTR, ceftriaxone; CIP, ciprofloxacin; GEN, gentamicin; TZP, piperacillin and tazobactam; TMP/SMX, trimethoprim/sulfamethoxazole.

### Evaluation of Retrieved Stents

In total, only 75 stents from 70 patients were retrieved at follow-up and analyzed. Two stents were retrieved during surgery for scheduled oncological resection. The degree of stent occlusion did not associate with early cholangitis (43.4% vs. 55.6%, *p* = 0.719), bile composition, positive bile culture at first visit (26.4% vs. 33.3%, *p* = 0.696) or at second visit (77.6% vs. 75%; *p* = 1.000), or adverse events during follow-up (14% vs. 14.3%, *p* = 1).

## Discussions

Biliary plastic stent dysfunction causing cholangitis increases in frequency with time and can represent a serious adverse event, but optimal timing for stent revision has not been determined. In this prospective study, we have found that early cholangitis after biliary plastic stenting is frequent, and that the risk is higher in those patients with a positive bile culture at the index procedure.

The disruption of normal biliary sphincter function leads to alterations in bile flow and composition, colonization, and potential clinically relevant infection due to enteric bacteria. Placement of a long indwelling foreign object such as a plastic stent leads to rapid biofilm formation and represents a predictable cause of recurrent obstruction and infection. Furthermore, instrumentalization of the biliary tract during ERCP and subsequent contamination with multidrug resistant microbes associated with duodenoscopes is of continued concern. 16 Recent literature highlights the high microbial burden associated with biliary stenting and its role in post-ERCP cholangitis. A large retrospective study 17 reported positive bile cultures in over 90% of ERCP procedures performed for suspected cholangitis, with Enterococcus spp., Klebsiella spp., and E. coli being the most frequently isolated organisms. The same study emphasized the reduced susceptibility of these pathogens to fluoroquinolones, especially in patients with prior sphincterotomy. However, while existing data focus largely on microbiological profiles in established cholangitis, 18 our study is among the first to evaluate the predictive value of bile colonization at the time of stent insertion.

Biliary PS represents the standard of care at the index ERCP procedure in cases of complex hilar strictures and early cholangitis severely affects the outcome of patients and their oncological management leading to repeated interventions, treatment delay, and potentially fatal outcome. Therefore, we strived to ascertain all relevant risk factors associated with a clinically relevant outcome defined in our study as cholangitis before scheduled stent exchange at 3 months. We analyzed patient, procedure, and management related factors in order to exclude confounders and analyzed bile composition and microbiological profile at two consecutive visits in order to understand the changes in the immune and biochemical environment brought about through sphincter disruption and stent placement. Furthermore, we attempted to retrieve the stents and quantify the degree of luminal obstruction, allowing for correlation between microbiological, biochemical, and mechanical indicators of early stent dysfunction. Positive bile cultures from samples extracted at the index ERCP procedure were the only statistically significant factor associated with our primary outcome of interest. The results of our study therefore suggest that routine bile sampling during ERCP may provide clinically relevant information for postprocedural management.

Our bile composition data add another dimension to the understanding of stent-related cholangitis. The significant post-stenting rise in biliary IgA and IgG concentrations is consistent with a sustained mucosal inflammatory response to the indwelling foreign body. More intriguing is the observation that biliary cholesterol levels at follow-up were significantly lower in patients who developed early cholangitis (median 20.2 vs. 66.65 mg/dL, *p* = 0.005). Although biliary cholesterol is a key factor for stent occlusion by sludge, its apparent inverse correlation with cholangitis risk suggests that in our predominantly malignant cohort, infectious episodes are not mainly driven by lipid and cholesterol occlusion. These findings are exploratory, the sample size available for bile composition analysis was limited ( *n* = 41), and so our findings need to be interpreted cautiously, but they highlight the potential value of bile biochemistry as a complementary risk-stratification tool alongside microbiological culture results.

These observations gain further relevance considering the pooled data from randomized controlled trials (RCTs) and observational studies, which show an overall cholangitis incidence of 2.5% in all-comers and 1.9% in first-time ERCP patients. However, in our cohort, the incidence of early cholangitis post-plastic stenting was significantly higher, reaching 34.6% within a median of 33 days. There are several reasons which may account for this difference: the predominance of complex hilar strictures in our cohort, differences in criteria employed for diagnosing cholangitis, overdiagnosis of cholangitis when using TG18 criteria in clinically asymptomatic patients, as well as our design which included routine lab work which might overestimate the prevalence of cholangitis in malignant stenosis.

On the other hand, the results also highlight the high risk nature of plastic stent placement in certain populations and suggests that the standard incidence rates may significantly underestimate early post-ERCP infection risk in patients with pre-existing biliary colonization. Meta-analyses comparing plastic and metal stents have shown that metal stents do not increase postoperative complications and may be preferable, particularly in patients receiving neoadjuvant therapy or scheduled for delayed surgery. 19 Our findings support this approach, as the high early complication rate with plastic stents may outweigh their short-term cost benefit in selected high-risk groups.

The present study raises the hypothesis that patients with a positive bile culture at the index ERCP may represent a higher risk group who could benefit from earlier stent revision. In order to validate this hypothesis, our group is currently conducting a randomized controlled study to test a personalized post ERCP follow-up strategy based on microbiological risk assessment: NCT07379749. Furthermore, our data raise questions regarding the effectiveness of systemic antibiotic therapy after successful biliary drainage. While antibiotics remain critical in acute cholangitis, their role in preventing subsequent episodes post-ERCP appears limited, especially considering persistent colonization and antibiotic resistance. Thus, mechanical source control via timely stent exchange may be more effective than extended antibiotic use in culture-positive patients.

This study has several limitations. It was conducted in a single tertiary referral center, which may affect the generalizability of findings. The high proportion of both malignant indications and baseline cholangitis in our cohort represents a referral center bias, potentially limiting the generalizability of our findings. The rate of dropout was acceptable but still represents a potential source of bias. Although we minimized contamination by flushing the sphincterotome lumen with sterile saline before cannulation and collecting bile before contrast injection, low-level contamination affecting the validity our results cannot be excluded. We also did not employ advanced molecular diagnostics, such as 16S rRNA sequencing, which has been shown to detect bacterial species not identified by conventional culture methods. 20 Furthermore, we based out definition of cholangitis on TG18 criteria and these may overestimate the rate of cholangitis at index procedures and scheduled follow-up, but this is a common limitation in studies assessing cholangitis. Additionally, our protocol included postprocedural blood work at 6 h and a telephonic follow-up, which may have captured the subclinical episodes of cholangitis which might have not been otherwise identified in standard clinical practice, therefore contributing to the higher incidence observed, in comparison to published rates.

In conclusion, early cholangitis after biliary plastic stenting was frequent in the study cohort and was associated with positive bile culture at index ERCP. Our findings suggest that bile sampling during index ERCP may identify patients who present a higher risk for early cholangitis. Prospective interventional studies are required to determine whether bile culture during initial plastic stenting can improve personalized care through early stent revision in selected cases.
